# Therapeutic targeting at genome mutations of liver cancer by the insertion of HSV1 thymidine kinase through Cas9-mediated editing

**DOI:** 10.1097/HC9.0000000000000412

**Published:** 2024-03-18

**Authors:** Muhamuda Kader, Wei Sun, Bao-Guo Ren, Yan-Ping Yu, Junyan Tao, Lesley M. Foley, Silvia Liu, Satdarshan P. Monga, Jian-Hua Luo

**Affiliations:** 1 Department of Pathology, University of Pittsburg School of Medicine, Pittsburgh, Pennsylvania, USA; 2 Pittsburgh Liver Research Center at Pittsburgh Liver Institute, Animal Imaging Center, University of Pittsburg School of Medicine, Pittsburgh, Pennsylvania, USA; 3 University of Pittsburgh School of Medicine, Pittsburgh, Pennsylvania, USA

## Abstract

**Background:**

Liver cancer is one of the most lethal malignancies for humans. The treatment options for advanced-stage liver cancer remain limited. A new treatment is urgently needed to reduce the mortality of the disease.

**Methods:**

In this report, we developed a technology for mutation site insertion of a suicide gene (herpes simplex virus type 1- thymidine kinase) based on type II CRISPR RNA-guided endonuclease Cas9-mediated genome editing to treat liver cancers.

**Results:**

We applied the strategy to 3 different mutations: S45P mutation of catenin beta 1, chromosome breakpoint of solute carrier family 45 member 2-alpha-methylacyl-CoA racemase gene fusion, and V235G mutation of SAFB-like transcription modulator. The results showed that the herpes simplex virus type 1-thymidine kinase insertion rate at the S45P mutation site of catenin beta 1 reached 77.8%, while the insertion rates at the breakpoint of solute carrier family 45 member 2 – alpha-methylacyl-CoA racemase gene fusion were 95.1%–98.7%, and the insertion at V235G of SAFB-like transcription modulator was 51.4%. When these targeting reagents were applied to treat mouse spontaneous liver cancer induced by catenin beta 1^S45P^ or solute carrier family 45 member 2-alpha-methylacyl-CoA racemase, the mice experienced reduced tumor burden and increased survival rate. Similar results were also obtained for the xenografted liver cancer model: Significant reduction of tumor volume, reduction of metastasis rate, and improved survival were found in mice treated with the targeting reagent, in comparison with the control-treated groups.

**Conclusions:**

Our studies suggested that mutation targeting may hold promise as a versatile and effective approach to treating liver cancers.

## Graphical abstract

**Figure:**
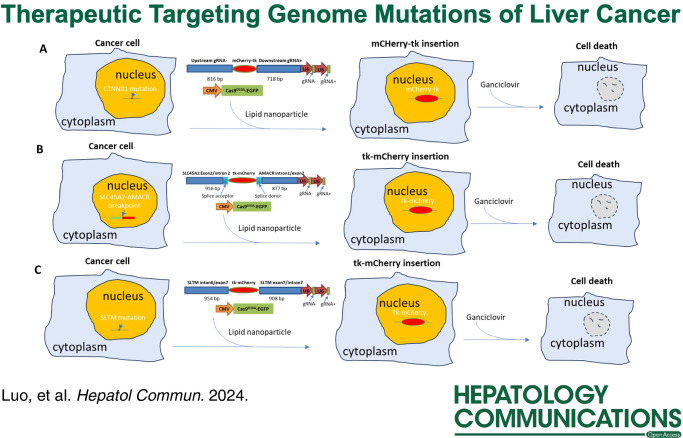

## INTRODUCTION

Liver cancer is one of the most lethal malignancies for humans. In 2022, 29,380 people in the United States died from liver cancer.^1^ Worldwide, mortality for liver cancer reached 830,180 in 2019.^2^ HCC has been the dominant liver cancer type, accounting for over 90% of all liver cancers. The most effective treatments for HCC are surgical resection and liver transplantation.^3^ However, these options are limited to early-stage liver cancer. For intermediate stages of HCC, transarterial radioembolization or transarterial chemoembolization has been employed with variable short-term successful rates. Long-term survival, however, remains elusive. For late-stage HCC, there is no effective treatment. The median survival time for late-stage HCC ranges from 6.1 to 10.3 months.^4^ Thus, an effective treatment for unresectable HCC is urgently needed to reduce the mortality of the disease.

Numerous genetic alterations have been discovered in liver cancer cells, including single-nucleotide mutations,^5^ genome deletion/amplification,^6^,^7^ chromosome rearrangement, and gene fusion generation.^8^,^9^,^10^,^11^,^12^,^13^ These genomic alterations underlie the development of HCC. A previous study has shown that chromosome rearrangement in human cancers is targetable through type II CRISPR RNA-guided endonuclease Cas9 (Cas9) genomic editing.^14^ By insertion of the herpes simplex virus type 1(HSV1)-thymidine kinase (tk) gene into the breakpoint of fusion gene mannosidase alpha class 2A member 1-FER or transmembrane protein 135-CCDC67 and the application of prodrug ganciclovir, partial remission of xenografted cancers was achieved in mice. In this report, we developed a strategy for targeting single-nucleotide mutation and gene fusion in the HCC genome to treat spontaneous and xenografted liver cancers. The results showed that the treatment significantly reduced the tumor burden, decreased metastasis/invasion, and improved animal survival.

## METHODS

### Cell lines

The cell lines used in the study were purchased from the American Type Culture Collection (ATCC, Manassas, Virginia) and were cultured and maintained following the manufacturer’s recommendations. They were authenticated every 6 months and were free of mycoplasma.

### Construction of vectors

To construct catenin beta 1 (CTNNB1) expression vector, cDNA of CTNNB1 was obtained by PCR using AccuPrime™ Pfx DNA Polymerase (Invitrogen) with a pair of primer (gtcgacCACCATGGAGCAAAAGCTCATTTCTGAAGAGGACTTG/gcggccgcTTACAGGTCAGTATCAAAC) corresponding to the CDS region of CTNNB1 with c-myc tag from Origene Inc. in the following condition: 94°C for 1 minute, then 35 heating cycles at 95°C for 15 seconds, 65°C for 30 seconds, and 72°C for 10 minutes. The PCR product was then restricted with Sal1 and NotI, and ligated into the similarly digested pT3-EF1a vector. The 5′ untranslated sequence of CTNNB1 was obtained by PCR with a pair of primers (tttaaa AGGATACAGCGGCTTCTGCGCG/ gtcgacCACGCTGGATTTTCAAAACAG on cDNA obtained from a liver organ donor in the same conditions as mentioned above. The PCR product was digested with Dra1 and Sal1 and ligated to the similarly restricted vector created from the first step to create a pT3-EF1a-CTNNB1 vector. To create a S45P mutation, 2-step mutational PCRs were performed: a PCR was performed with mutagenic primers tttaaaAGGATACAGCGGCTTCTGCGCG/GCCTTTACCACTCAGAGgAGGAGCTGTGGTAGT using AccuPrime™ Pfx DNA Polymerase in the following condition: 94°C for 1 minute, then 35 heating cycles at 95°C for 15 seconds, 65°C for 30 seconds, and 72°C for 10 minutes. A separate PCR was performed using primers CACTACCACAGCTCCTcCTCTGAGTGGTAAAGGCAA TC/ gcggccgcTTACAGGTCAGTATCAAAC. A final PCR was then performed on the mixture of 2 PCR products using primers tttaaaAGGATACAGCGGCTTCTGCGCG and gcggccgcTTACAGGTCAGTATCAAAC to obtain the mutant cDNA. The mutant product was digested with Dra1 and Not1 and ligated into the similarly digested pT3-EF1a vector to create the pT3-EF1a-CTNNB1^S45P^ construct.

To construct a CTNNB1-HSV1-tk-RA-gRNA-/gRNA+ vector, a chimera mCherry-2A-HSV1-tk coding sequence was synthesized. An 815 bp 5’ cDNA from CTNNB1 was then ligated to the upstream of the synthetic chimera DNA. Subsequently, a 718 bp 3’ cDNA from CTNNB1 was ligated downstream to the synthetic chimera DNA. The donor vector was completed by insertion of U6-gRNA-U6-gRNA+ synthetic DNA block downstream to the 3’ cDNA of CTNNB1.

To construct pT3-EF1a-SLC45A1^exon1-2^-breakpoint intron-alpha-methylacyl-CoA racemase (AMACR)^exon2-6^, the 4031 bp DNA that included exons 1-2 of solute carrier family 45 member 2 (SLC45A2), followed by 1117 bp breakpoint intron and exons 2-6 of AMACR was chemically synthesized (BioBasic, Inc). The construct was then ligated into pT3-EF1a. To construct solute carrier family 45 member 2 – alpha-methylacyl-CoA racemase (SLC45A2-AMACR)-HSV1-tk-mCherry-gRNA vector, a fragment of 4167 bp DNA containing 956 bp from exon 2 and intron 2 of SLC45A2, splice acceptor (100 bp) of intron 2 of SLC45A2, a chimera coding sequence of HSV1-tk-2A-mCherry, splice donor (100 bp) of intron 3 of AMACR, 877 bp DNA from intron 2 and exon 3 of AMACR was chemically synthesized. A unit of U6-gRNA-U6-gRNA+ was then inserted downstream into the synthetic DNA to complete the construction. To construct pSLTM-mCherry-tk-gRNA vector, a 4796 bp donor DNA fragment was synthesized to include a 954-bp fragment of exon7 and intron 7 of SAFB-like transcription modulator (SLTM), a chimera coding sequence for mCherry-2A-HSV1-tk, a 908-bp fragment of intron 7 and exon 8 of SLTM, followed by a U of U6-gRNA-U6-gRNA+, was chemically synthesized. Cas9^D10A^-enhanced green fluorescent protein (EGFP) construct was obtained from Addgene, inc.

### *In vitro* Cas9 target cleavage assays

gRNA DNA sequence plus scaffold DNA sequence for + or − DNA strand were amplified from the all-in-one vector with the following primers: GGCCAGTGAATTGTAATACGACTCACTATAGGGAGGCGG AAAGGCAATCCTGAGGAAG/AAAAAAAGCACCGACTCGGTGCCACTTTTTC for gRNA+ template of CTNNB1, GGCCAGTGAATTGTAATACGACTCACTATAGGGAGGCGGTCTACCACAGCTCCTCCTCTG/ AAAAAAAGCACCGACTCGGTGCCACTTTTTC for gRNA− template of CTNNB1, GGCCAGTGAATTGTAATACGACTCACTATAGGGAGGCGGGGTGCTAAACTTTTTCGTGA/AAAAAAAGCACCGACTCGGTGCCACTTTTTC for gRNA+ template of SLC45A2-AMACR, GGCCAGTGAATTGTAATACGACTCACTATAGGGAGGCGGGAGAGCTCCCATTTTCCTCC/AAAAAAAGCACCGACTCGGTGCCACTTTTTC for gRNA− template of SLC45A2-AMACR, GGCCAGTGAATTGTAATACGACTCACTATAGGGAGGCGG CTGTGTGATCAGCCTCAGCT/ AAAAAAAGCACCGACTCGGTGCCACTTTTTC for gRNA+ template of SLTM, and GGCCAGTGAATTGTAATACGACTCACTATAGGGAGGCGG AGATGGAAGCTAATGCGACT/AAAAAAAGCACCGACTCGGTGCCACTTTTTC for gRNA− template of SLTM. The PCR products were in vitro transcribed using an In Vitro Transcription kit from Ambion, CA, to obtain gRNA+ and gRNA− products. Cleavage assays were performed at 25°C for 10 minutes and then 37°C for 1 hour under the following conditions: 1x Cas9 nuclease reaction buffer, 30 nM gRNA 3 nM DNA template, and 30 nM Cas9 Nuclease, *S. pyogenes*. The cleaved DNA was visualized in 1% agarose gel electrophoresis.

### Fluorescence-activated cell sorting (FACS) analysis of apoptotic cells

The assays were previously described^15^,^16^,^17^,^18^,^19^,^20^,^21^,^22^,^23^. Briefly, the cells treated with Ad-Cas9^D10A^-EGFP/ Ad-mCherry-tk Ad-CTNNB1-mCherry-tk-gRNA or Ad-Cas9^D10A^-EGFP /ad-SLC45A2-AMACR (SLAM)-HSV1-tk-mCherry-gRNA or pCas9^D10A^-EGFP /pSLTM-mCherry-HSV1-tk were trypsinized and washed twice with cold PBS. The cells were analyzed at the fluorescence emission at 610 nm (mCherry) and 509 nm (EGFP), respectively. The negative control, cells without treated reagents in the incubation medium, was used to set the background for the acquisition. WinMDI 2.9 software (freeware from Joseph Trotter) was used to analyze the data.

### Mice and hydrodynamic tail vein injections

All research was conducted in accordance with both the Declarations of Helsinki and Istanbul. The animal protocols have been approved by the Institutional Animal Care and Utility Committee of the University of Pittsburgh (22091650). Hydrodynamic tail vein injections were performed as described previously^24^,^25^. Briefly, first, Pten^tm1Hwu/J^ mice of which exon 5 of the Pten gene flanked by loxP sites were treated with adeno-associated virus-cre (1×10^10^ PFU) through intraperitoneal injection to create Pten knockout in most hepatocytes. Next, 20 μg of pT3-SLC45A2^exon1-2^-breakpoint intron-AMACR^exon2-6^-FLAG, along with the sleeping beauty transposase (SB) in a ratio of 25:1 were diluted in 2 ml of normal saline (0.9% NaCl), filtered through 0.22 μm filter (Millipore), and injected into the lateral tail vein in 5 to 7 seconds. Mice were housed, fed, and monitored in accordance with the protocols approved by the Institutional Animal Care and Use Committee at the University of Pittsburgh School of Medicine. A similar hydrodynamic tail vein injection was performed with pT3-EF1a-CTNNB1S45P/MET proto-oncogene, receptor tyrosine kinase (HMET) injection except for 20 μg for pHMET and pT3-EF1a-CTNNB1^S45P^ each on FVB/NJ mice.

### Tumor growth and spontaneous metastasis

The sample size of tumor xenografting analysis was based on power analysis, assuming 90% survival for treated animals and 10% for control-treated. Complete blindness was applied in the animal study. The xenografting procedure was described^17^,^21^,^22^,^23^,^26^,^27^,^28^. Briefly, 5×10^6^ viable HUH7-CTNNB1, HUH7-CTNNB1^S45P^, HUH7, or HEPG2 cells, suspended in 0.2 mL of Hanks’ balanced salt solution (Krackeler Scientific, Inc., Albany, NY) were subcutaneously implanted in the abdominal flanks of severe combined immuno-deficiency (SCID) mice to generate one tumor per mouse. The breakdown of the treated groups is the following: 6 for HUH7 cells transduced with pT3-EF1a-CTNNB1^S45P^ and treated with pCTNNB1-mCherry-tk-gRNA/pCas9^D10A^-EGFP and ganciclovir; 6 for HUH7 cells transduced with pT3-EF1a-CTNNB1 and treated with pCTNNB1-mCherry-tk-gRNA/pCas9^D10A^-EGFP and ganciclovir (control); 6 for HUH7 cells transduced with pT3-EF1a-CTNNB1^S45P^ and treated pCTNNB1-mCherry-tk (no gRNA)/pCas9^D10A^-EGFP and ganciclovir (control); 6 for HUH7 cells transduced with pT3-EF1a-CTNNB1 and treated with pCTNNB1-mCherry-tk (no gRNA)/pCas9D10A-EGFP and ganciclovir (control). For SLC45A2-AMACR targeting, the distribution is the following: 10 mice for HEPG2 cells and treated with pSLAM-tk-mCherry-gRNA/pCas9^D10A^-EGFP and ganciclovir, 5 for HEPG2 cells and treated with pCas9^D10A^-EGFP and ganciclovir, 5 for HEPG2 cells and treated with pSLAM-tk-mCherry-gRNA and ganciclovir, 5 for HUH7 cells and treated with pSLAM-tk-mCherry-gRNA/pCas9^D10A^-EGFP and ganciclovir, 5 for HUH7 and treated with pSLAM-tk-mCherry (no gRNA) /pCas9^D10A^-EGFP and ganciclovir. For SLTM mutation targeting, the distribution is the following: 5 mice for HUH7 and treated with pSLTM-mCherry-tk-gRNA/pCas9^D10A^-EGFP and ganciclovir, 5 mice for HUH7 and treated with pSLTM-mCherry-tk-gRNA and ganciclovir, and 5 mice for HUH7 and treated with pSLTM-mCherry-tk-gRNA and ganciclovir. Mice were observed daily, and their body weight and tumor size were recorded weekly. The format of the tumor measurement is based on ellipsoid volume: π*length*width*depth/6. Three weeks after xenografting, these mice were treated with the indicated cocktail of in vivo-JetPEI with the proportion based on the recommendation from the manufacturer (Genesee Scientific, Inc.) and ganciclovir (80 mg/kg) 3 times a week through tail vein injection. After 42 days, mice in the treatment groups were killed, and necropsies were performed. For mice treated with control reagents, necropsies were performed when mice died from the xenografted cancers. The protocol of animal experiments is approved by the Institutional Review Board of the University of Pittsburgh.

### Methods of euthanasia

CO_2_ euthanasia was performed at the end points of the animal studies. Briefly, without pre-charging the chamber, the animals were placed in the chamber and introduced to 100% CO_2_ at a rate of 30%–70% of the chamber volume per minute. After the animals became unconscious, the flow rate was increased to minimize the time to death. When the rodent was found to have respiratory arrest and to have faded eye color, the carcass was then removed from the cage. Cervical dislocation was used as a secondary method to euthanize the animals if CO_2_ euthanasia was inappropriate.

### Magnetic resonance imaging

Mice were anesthetized through a nose cone with 1–2% isoflurane and O_2_; they were then positioned on an animal bed with the abdomen secured to reduce motion artifacts and placed in the scanner. Respiration rate was monitored, and body temperature was maintained using a warm air heating system (SA Instruments, New York, NY, USA). MRI was performed on a 7T/30-cm AVIII spectrometer (Bruker Biospin, Billerica, MA) equipped with a 12-cm gradient set and using a 40-mm quadrature RF volume coil and Paravision 6.0.1. A T_2_-weighted RARE sequence was used to visualize the liver tumors, with the following parameters: repetition time (TR)/echo time (TE) = 5000/24 ms, field of view (FOV) of 35 mm^2^, acquisition matrix=256×256, 30–47 slices (depending on liver tumor size) with a slice thickness of 0.7 mm, 8 averages, and a RARE factor=8. The tumor volume was calculated based on the aggregates of tumor nodule volumes using the DSI studio volume calculation function.

## RESULTS

### Targeting the CTNNB1^S45P^ mutation in vitro and in vivo

**FIGURE 1:**
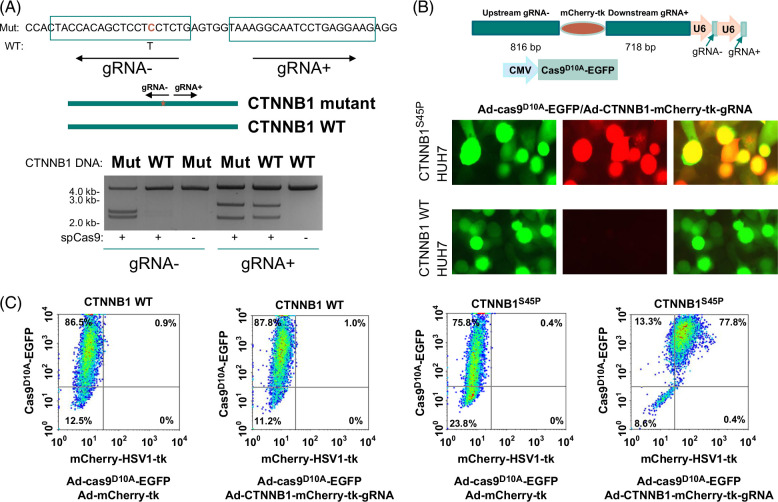
Targeting S45P mutation of CTNNB1. (A) gRNA design and in vitro cleavage of mutant DNA by SpCas9. Top: sequence with C. 133T > C mutation from exon 2 of CTNNB1. The gRNA design is indicated by a rectangular bracket. The mutation position is indicated in red. The corresponding wild-type nucleotide is indicated. Bottom: Cleavage of mutant DNA by gRNA−, while cleavage of WT DNA by gRNA− negligible. (B) HUH7 cells transduced with CTNNB1^S45P^ showed insertion/expression of mCherry-HSV1-tk, while HUH7 cells transduced with CTNNB1 showed no insertion/expression when treated with ad-Cas9^D10A^-EGFP/ad-CTNNB1-mCherry-tk-gRNA. Top: diagrams of targeting constructs; Bottom: Representative images of EGFP and mCherry fluorescence. (C) Induction of expression of mCherry-HSV1-tk in HUH7 cells transduced with CTNNB1^S45P^ but not with CTNNB1. Abbreviations: CTNNB1, catenin beta 1; cas9, type II CRISPR RNA-guided endonuclease Cas9; EGFP, enhanced green fluorescence protein; HSV1, herpes simplex virus type 1; SpCas9, Staphylococcus pyogenes cas9; tk, thymidine kinase; WT, wild-type.

CTNNB1 mutations are frequent in the genome of HCC and can range from 26 to 37% of all HCC cases.^29^ Among the mutation profiles of CTNNB1, the mutation that converts serine at position 45 to proline is one of the frequent mutations in human HCC samples. Such mutation has been shown to drive HCC development in mice.^30^ To investigate whether S45P mutation is targetable by Cas9-mediated genome editing, we designed a pair of gRNAs specific for thymidine to cytosine mutation at the position of 133 of the coding sequence of CTNNB1, which results in the conversion of serine to proline. To test the specificity of the gRNA, the gRNA− and staphylococcus pyogenes cas9 were applied to cleave a 4.8-kb mutant (C. 133T>C) CTNNB1 cDNA in vitro (Figure 1A). The reaction cleaved the mutant DNA into 2.3 and 2.5-kb fragments, respectively, while the same application yielded no impact on the wild-type cDNA. On the other hand, the application of gRNA+ and staphylococcus pyogenes cas9 resulted in the cleavage of both mutant and wild-type CTNNB1 templates into fragments of 2.2 and 2.6 kb. These results suggest that gRNA− is highly specific for CTNNB1 mutants.

HSV1-tk is a thymidine kinase from HSV1. It phosphorylates thymidine to generate thymidine monophosphate, which is in turn, used as a substrate to produce thymidine triphosphate for DNA synthesis. However, HSV1-tk contains subtle differences in substrate specificity versus its mammalian counterparts:^31^ it phosphorylates ganciclovir, a prodrug for Herpes treatment, into ganciclovir-monophosphate, which is subsequently converted into ganciclovir triphosphate. Ganciclovir triphosphate will be utilized in the host cells as DNA building blocks and cause cell death due to faulty DNA structure.^32^ Normal mammalian thymidine kinase lacks substrate specificity of ganciclovir. Thus, ganciclovir has minimal impact on mammalian cells that do not contain HSV1-tk. Next, we constructed a recombinant adenovirus containing these gRNAs and a donor unit that contained sequences mCherry-HSV1-tk sandwiched by cDNA sequences upstream and downstream of the targeted sequence (Figure 1B). The cassette of mCherry-HSV1-tk contains no promoter but retains the ribosome-binding site and an intact ATG translation start site. Thus, mCherry-HSV1-tk was not expressed unless the cassette was inserted into an active transcribed position in the genome. This recombinant adenovirus was then co-infected with a recombinant adenovirus expressing Cas9^D10A^-EGFP into the HUH7 cells that were transduced with the pT3-EF1a-CTNNB1^S45P^ expression vector. As shown in Figures 1B and C, the co-introduction of ad-Cas9^D10A^-EGFP and ad-CTNNB1-mCherry-tk-gRNA induced strong expression of mCherry-HSV1-tk in HUH7 cells expressing CTNNB1^S45P^, with expression frequency reaching 77.8% of the infected cells. On the other hand, the expression of mCherry-HSV1-tk was minimal when HUH7 cells were transduced with the wild-type CTNNB1 cDNA construct. In the absence of gRNA, little expression of mCherry-HSV1-tk was detected in CTNNB1^S45P^-transduced HUH7 cells. These results suggested that the insertion of mCherry-HSV1-tk into the cancer genome by these reagents was CTNNB1^S45P^ mutation-dependent.

**FIGURE 2:**
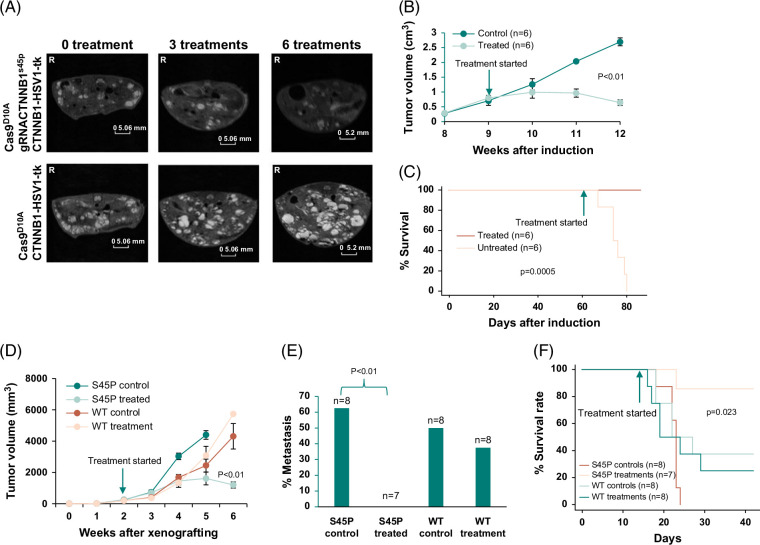
Therapeutic effect of targeting S45P of CTNNB1. (A) Magnetic resonance images of CTNNB1^S45P^/HMET–induced liver cancer. Top panel: Representative MRI images of a mouse treated with pCas9^D10A^-EGFP/pCTNNB1-mCherry-tk-gRNA (treated). Bottom panel: Representative MRI images of a mouse treated with pCas9^D10A^-EGFP/pCTNNB1-mCherry-tk (control). (B) Accumulated liver cancer growth by image analysis of treated and control groups. (C) Kaplan-Meier analysis of mice survival from the treatment and control groups. (D) Treatment targeting S45P of CTNNB1 reduced the tumor burden of xenografted HUH7-CTNNB1^S45P^ but not HUH7-CTNNB1 in SCID mice. (E) Treatment targeting S45P of CTNNB1 reduced metastasis of xenografted HUH7-CTNNB1^S45P^ in SCID mice. (F) Treatment targeting S45P of CTNNB1 reduced mortality of SCID mice xenografted with HUH7-CTNNB1^S45P^ cells. Abbreviations: CTNNB1, catenin beta 1; cas9, type II CRISPR RNA-guided endonuclease Cas9; EGFP, enhanced green fluorescence protein; HMET, MET proto-oncogene, receptor tyrosine kinase; MET proto-oncogene, receptor tyrosine kinase; SCID, severe combined immuno-deficiency.

To investigate whether these genome-targeting reagents have a therapeutic effect, pT3-CTNNB1^S45P^ was hydrodynamically injected into the tail vein of FVB/NJ mice along with pT3-HMET and pSB (plasmid sleeping beauty) to induce spontaneous liver cancer. As shown in Figure 2A, numerous small liver cancer nodules began to appear in MRI 2 months after the injection. In the ninth week, a cocktail of genome targeting reagents, including pCas9^D10A^-EGFP and pCTNNB1-mCherry-tk-gRNA, mixed with in vivo-JetPEI delivery reagents were injected into the tail vein of the animals 3 times a week for HSV1-tk insertion into the cancer genome. This was coupled with an i.p. injection of prodrug ganciclovir for cancer treatment. In vivo-JetPEI has been widely used as a DNA/RNA delivery reagent for gene therapy and has shown high delivery efficiency.^19^,^20^,^21^,^33^,^34^,^35^,^36^,^37^ The progression of the liver cancers was monitored through MRI imaging in a weekly fashion. As shown in Figures 2A and B, there is a gradual reduction of tumor volume after 4 weeks of treatment. In contrast, the control-treated (pCas9^D10A^-EGFP/pCTNNB1-mCherry-tk, no gRNA) group experienced a 3.9-fold increase in tumor volume in the same period. All control-treated animals died in 90 days after the induction of liver cancer, while all the animals treated with the therapeutic reagents survived beyond this period (Figure 2C, *p* = 0.0004).

To investigate whether CTNNB1^S45P^-targeting reagents also worked similarly in human cancers that contained CTNNB1^S45P^ mutation, HUH7 cells were transfected with pT3-EF1a-CTNNB1^S45P^-FLAG. HUH7 cells positive for CTNNB1^S45P^-FLAG expression were xenografted into the subcutaneous region of severe combined immunodeficiency mice. When the xenografted cancers reached an average of 229 mm^3^, the CTNNB1^S45P^-targeting therapeutic reagents were applied through the tail vein injection as described above. As shown in Figure 2D, the treatment of the CTNNB1^S45P^ targeting limited the growth of HUH7 cells harboring the mutation, while the control-treated groups experienced exponential growth of the cancer. On the other hand, HUH7 cells harboring the wild-type CTNNB1 did not respond to the CTNNB1^S45P^ targeting therapeutic reagents, as the tumor volumes of the treated groups did not show appreciable reduction versus those of the controls. All seven animals xenografted with HUH7 cells containing CTNNB1^S45P^ and treated with including pCas9^D10A^-EGFP and pCTNNB1-mCherry-tk-gRNA had no incidence of metastasis/invasion (Figure 2E). On the other hand, the CTNNB1^S45P^ HUH7–xenografted animals treated with control reagents had a 62.5% (5/8, *p* = 0.03) rate of metastasis/invasion. Animals xenografted with HUH7 containing wild-type CTNNB1 had similar rates of metastases (4/8 vs 3/8, *p* = 1). All animals xenografted with HUH7 harboring CTNNB1^S45P^ and treated with the control therapeutic reagents died less than 4 weeks after the tumor xenografting, while 6/7 animals treated with CTNNB1^S45P^-targeting reagents survived through 42 days. In contrast, the mortality rate of animals xenografted with HUH7 cells harboring the wild-type CTNNB1 showed minimal difference in mortality (5/8 versus 6/8 mortality) between the treated and the control group. These results suggested that mutation targeting at CTNNB1^S45P^ was highly specific and was mutation-dependent.

### Targeting the breakpoint of SLC45A2-AMACR

**FIGURE 3:**
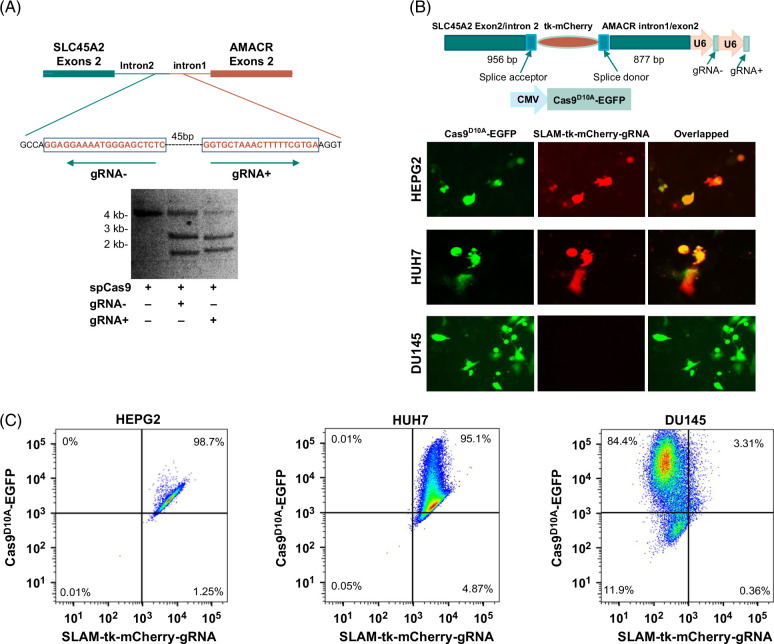
Targeting the chromosome breakpoint of SLC45A2-AMACR. (A) gRNA design and in vitro cleavage of mutant DNA by SpCas9. Top: Diagram of breakpoint region between SLC45A2 intron 2 and AMACR intron 1. The gRNA design is indicated in red and enclosed by a rectangle. Bottom: Cleavage of sequence containing the breakpoint by spCas9 with gRNA+ or gRNA-. (B) SLC45A2-AMACR-positive HUH7 and HEPG2 cells showed insertion/expression of HSV1-tk-mCherry, while SLC45A2-AMACR-negative DU145 cells showed no expression of HSV1-tk-mCherry when treated with ad-Cas9^D10A^-EGFP/ad-SLAM-mCherry-tk-gRNA. Top: diagrams of targeting constructs; Bottom: Representative images of EGFP and mCherry fluorescence. (C) Flow cytometry analysis of HEPG2, HUH7, and DU145 cells when treated with ad-Cas9^D10A^-EGFP/ad-SLAM-mCherry-tk-gRNA. Abbreviations: AMACR, alpha-methylacyl-CoA racemase; cas9, type II CRISPR RNA-guided endonuclease Cas9; EGFP, enhanced green fluorescence protein; HSV1, herpes Simplex Virus Type 1; SLAM, SLC45A2-AMACR; SLC45A2, solute carrier family 45 member 2; SpCas9, staphylococcus pyogenes cas9; tk, thymidine kinase.

SLC45A2-AMACR gene fusion occurs frequently in human cancers. The rate of occurrence of this fusion gene in HCC reaches 78.6%.^38^ Interestingly, all HCC cell lines positive for SLC45A2-AMACR had identical breakpoints for the fusion in their genomes. Introduction of SLC45A2-AMACR into mouse liver along with somatic knockout of Pten has been shown to induce spontaneous liver cancer in a short period of time.^38^ SLC45A2-AMACR translocates the racemase domain of AMACR from mitochondria to cytoplasm and induces amplification of ERK2/MEK signaling.^38^ To investigate whether the breakpoint of SLC45A2-AMACR is targetable by Cas9-mediated insertion of HSV1-tk, a pair of gRNA was designed to direct the cutting in the breakpoint region of the cancer genome by Cas9. As shown in Figure 3A, the application of gRNA− to the digestion mix of staphylococcus pyogenes cas9 cut the breakpoint containing DNA fragment of 4.1 kb into 2.6 and 1.5 kb, while the gRNA+ cleaved the same fragment into 2.5 and 1.6 kb, matching the expected cutting position generated by these gRNAs. To investigate these gRNA-induced insertions of HSV1-tk into the breakpoint region of SLC45A2-AMACR in vivo, a donor-gRNA construct was generated by ligating a 956 bp DNA corresponding to exon 2 and intron 2 of SLC45A2 with tk-mCherry, followed by 877 bp of intron 1 and exon 2 from AMACR (Figure 3B). The construct was packaged into ad5 to create a recombinant adenovirus (ad-SLAM-tk-mCherry-gRNA) containing the donor cassette and expressing the gRNAs. Ad-SLAM-tk-mCherry-gRNA was then applied to co-infect with a recombinant adenovirus that expressed Cas9^D10A^-EGFP into HEPG2, HUH7, and DU145 cells. As shown in Figures 3B and C, the co-infection induced strong expression of HSV1-tk-mCherry in SLC45A2-AMACR-positive HEPG2 and HUH7 cells, with 98.7 and 95.1% of HEPG2 and HUH7 cells being positive for HSV1-tk-mCherry, respectively. In contrast, DU145, a cell line negative for SLC45A2-AMACR fusion, had only 3.3% positive for HSV1-tk-mCherry expression. These results suggested that the insertion of HSV1-tk-mCherry into the genome is specific to the breakpoint of SLC45A2-AMACR gene fusion.

**FIGURE 4:**
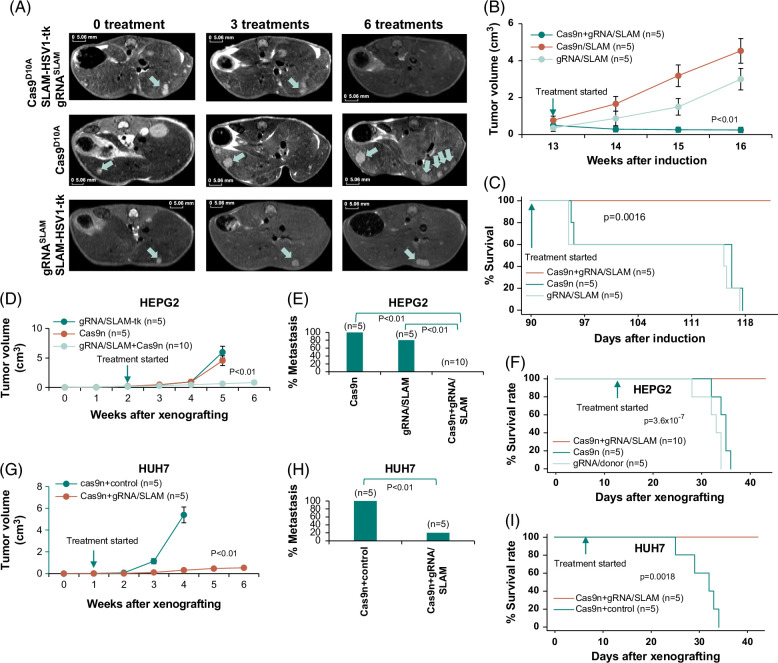
Therapeutic effect of targeting the chromosome breakpoint of SLC45A2-AMACR gene fusion. (A) Magnetic resonance images of SLC45A2-AMACR/Pten knockout–induced liver cancer. Top panel: Representative MR images of a mouse treated with pCas9^D10A^-EGFP/pSLAM-tk-mCherry-gRNA (treated). Middle panel: Representative MR images of a mouse treated with pCas9^D10A^-EGFP (control). Bottom panel: Representative MR images of a mouse treated with pSLAM-tk-mCherry-gRNA (control). Green arrows indicate the position of liver cancer in the image. (B) Accumulated liver cancer growth by image analysis of treated (Cas9n+gRNA/SLAM) and control groups (Cas9n or gRNA/SLAM). (C) Kaplan-Meier analysis of mice survival after treatment (Cas9n+gRNA/SLAM) or control treatment (Cas9n or gRNA/SLAM). (D) Treatment targeting the chromosome breakpoint of SLC45A2-AMACR reduced the tumor burden of HEPG2 xenografted tumor in SCID mice. (E) Treatment targeting the chromosome breakpoint of SLC45A2-AMACR reduced the rate of metastasis of HEPG2 xenografted tumors in SCID mice. (F) Treatment targeting the chromosome breakpoint of SLC45A2-AMACR reduced mortality of mice xenografted with HEPG2 tumor. (G) Treatment targeting the chromosome breakpoint of SLC45A2-AMACR reduced the tumor burden of HUH7 xenografted tumor in SCID mice. (H) Treatment targeting the chromosome breakpoint of SLC45A2-AMACR reduced the rate of metastasis of HUH7 xenografted tumors in SCID mice. (I) Treatment targeting the chromosome breakpoint of SLC45A2-AMACR reduced mortality of mice xenografted with HUH7 tumor. Abbreviations: AMACR, alpha-methylacyl-CoA racemase; cas9, type II CRISPR RNA-guided endonuclease Cas9; EGFP, enhanced green fluorescence protein; pCas9, plasmid cas9; SCID, severe combined immuno-deficiency; SLAM, SLC45A2-AMACR; SLC45A2, solute carrier family 45 member 2; tk, thymidine kinase.

To investigate whether targeting SLC45A2-AMACR is feasible in a spontaneous liver cancer model, we constructed an SLC45A2-AMACR cDNA with a full breakpoint intron to mimic the genome structure of SLC45A2-AMACR gene fusion. This intron-containing cDNA was constructed into a pT3-E1a vector to create pT3-SLC45A2^exon1-2^-breakpoint intron-AMACR^exon2-6^-FLAG. The construct was then hydrodynamically injected with pSB into the tail vein of pten^flox^ mice, where Pten was somatically disrupted in the hepatocytes through i.p. application of AAV8-cre. Spontaneous liver cancer was detected 12 weeks after the introduction of pT3-SLC45A2-breakpoint intron-AMACR-FLAG (Figure 4A). The treatment with SLC45A2-AMACR breakpoint targeting and Cas9^D10A^-EGFP expression constructs was applied using an in viv*o*-JetPEI delivery system the following week. As shown in Figures 4A and B, a gradual reduction of tumor burden was found for cancers treated with the constructs of pCas9^D10A^-EGFP and pSLAM-HSV1-tk-mCherry-gRNA. On the other hand, when the liver cancers were treated with pCas9^D10A^-EGFP or pSLAM-HSV1-tk-mCherry-gRNA alone, the tumor progressed significantly, reaching 18.2- (*p* < 0.01) and 12.1 (*p* < 0.01)-fold of the treatment group, respectively. All animals died from cancer in 4 weeks if not treated with the correct targeting reagents, while all the animals survived the same period when treated with the correct targeting reagents (*p* = 0.0016, Figure 4C).

To investigate whether the SLC45A2-AMACR genome-targeting reagents were also effective in human cancer cell lines that contained the gene fusion, HEPG2, which was positive for SLC45A2-AMACR, was xenografted to SCID mice subcutaneously. When the average tumor size of HEPG2 reached 167 mm^3^, a cocktail of in vivo-JetPEI containing the constructs of Cas9^D10A^-EGFP and pSLAM-HSV1-tk-mCherry-gRNA was injected into the tail vein of the mice 3 times a week. As shown in Figure 4D, the treatment reduced the tumor burden by an average of 9.5-fold (*p* < 0.01) in comparison with the pSLAM-HSV1-tk-mCherry-gRNA only controls or of 7.3-fold (*p*<0.01) with Cas9^D10A^-EGFP only controls. No incidence of metastasis or invasion was found for the animals treated with Cas9^D10A^-EGFP and pSLAM-HSV1-tk-mCherry-gRNA. In contrast, the incidence of metastasis/invasion reached 100% for Cas9^D10A^-EGFP only (*p* < 0.01) and 80% for pSLAM-HSV1-tk-mCherry-gRNA only control group (*p* < 0.05, Figure 4E). All mice in the control groups died less than 42 days after the xenografting, while all mice in the treatment group survived the same period (Figure 4F). Similar effects were seen with the constructs of pCas9^D10A^-EGFP and pSLAM-HSV1-tk-mCherry-gRNA on HUH7-xenografted SCID mice: The treatment reduced the tumor burden by an average of 17.6-fold (*p* < 0.01, Figure 4G) and decreased incidence of metastasis/invasion by 4-fold (*p* < 0.05, Figure 4H). The control-treated mice xenografted with HUH7 died less than 35 days after the xenografting, while all the mice in the treatment group survived through 42 days (*p* = 0.0018, Figure 4I). These results suggest that SLC45A2-AMACR genome-targeted treatment may be an effective approach to treating liver cancers.

### Targeting naturally occurring mutations of unknown significance

**FIGURE 5:**
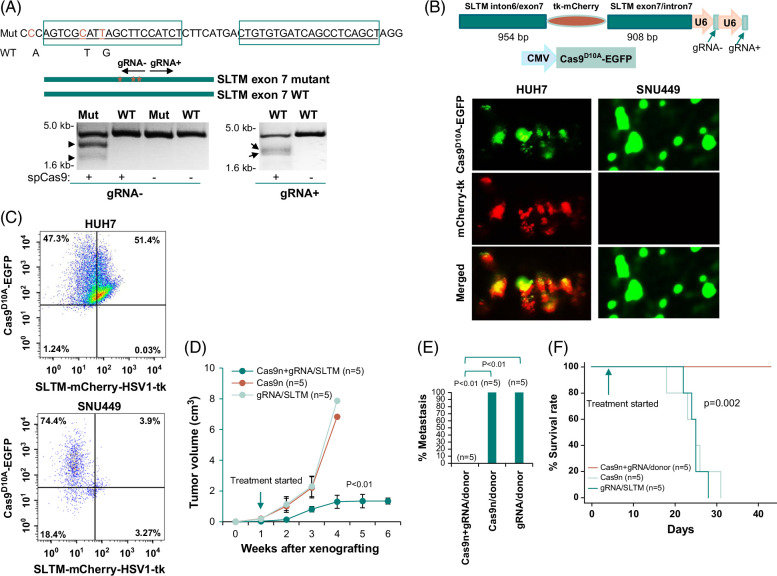
Targeting V235G mutation of SLTM in vitro and in vivo. (A) gRNA design and in vitro cleavage of mutant DNA by SpCas9. Top: sequence with mutations (Hg19- Chr15: 58899823, Hg19-Chr15: 58899830, and Hg19-Chr15: 58899833) in exon 7 of SLTM. The gRNA design is indicated by a rectangular bracket. The mutation position is indicated in red. The corresponding wild-type nucleotide is indicated. Bottom: Cleavage of mutant DNA by gRNA−, while cleavage of WT DNA by gRNA− negligible. Arrows indicate cleaved DNA fragments of the correct sizes. (B) HUH7 cells showed insertion/expression of mCherry-HSV1-tk, while SNU449, which was negative for the mutation, showed no insertion/expression when treated with ad-Cas9^D10A^-EGFP/pSLTM-mCherry-tk-gRNA. Top: diagrams of targeting constructs; Bottom: Representative images of EGFP and mCherry fluorescence. (C) Induction of expression of mCherry-HSV1-tk in HUH7 cells but not SNU449 cells. (D) Treatment targeting V235G of SLTM reduced the tumor burden of xenografted HUH7. (E) Treatment targeting V235G of SLTM reduced metastasis of xenografted HUH7 in SCID mice. (F) Treatment targeting V235G of SLTM reduced mortality of SCID mice xenografted with HUH7 cells. Abbreviations: cas9, type II CRISPR RNA-guided endonuclease Cas9; EGFP, enhanced green fluorescence protein; HSV1, herpes simplex virus type 1; SCID, severe combined immuno-deficiency; SLTM, SAFB-like transcription modulator; SpCas9, staphylococcus pyogenes cas9; tk, thymidine kinase; WT, wild-type.

The flexibility of Cas9-mediated genome targeting makes it feasible to target a wide variety of mutations. Many mutations in cancer cells may not be cancer drivers but nonetheless play important roles in assisting cancer development. The inclusion of these mutations in targeting not only increases the repertoire of targets against cancer cells but also provides a new approach to counter genome heterogeneity of liver cancer. To investigate the flexibility of mutation targeting, the exome and transcriptome of HUH7 cells were sequenced. Six hundred and fourteen single nucleotide variants (SNV) in the HUH7 genome were matched in both mRNA and exome levels and were identified as the pathological mutations defined in the COSMIC database. One of the SNVs occurred in a gene called SAFB-like transcription modulator (SLTM). The SNV was located in exon 7. The SNV converted valine at 235 of SLTM to glycine (C. 704 T>G, Hg19- Chr15: 58899823). Interestingly, 2 additional SNVs were found in a nearby region (C. 697A>G, HG19-Chr15: 58899830, and C. 694C>A, Hg19-Chr15: 58899833) of SLTM. The mutation of C. 704 T>G produced a potential new protospacer-adjacent motif sequence for Cas9. The mutations were found to be expressed abundantly at the mRNA level. These mutations are not known to be cancer-driving events. Thus, they are likely passenger mutations. To examine whether passenger mutation targeting by suicide gene insertion is also effective in treating cancer, we then designed a gRNA encompassing mutations C. 694C>A and C. 697A>G, and utilized the mutation C. 704 T>G as a part of the protospacer-adjacent motif sequence. As shown in Figure 5A, the gRNA specific for the mutations resulted in cleavage of mutant DNA but had no impact on the wild-type DNA. Next, we constructed a DNA fragment expressing these gRNAs and containing a promoterless mCherry-HSV1-tk sandwiched by 954 bp from intron 6 and the 5’ end of exon 7 of SLTM and 908 bp from the 3’ end of exon 7 and intron 7 of SLTM (Figure 5B). This construct was transfected into HUH7 cells that were infected with ad-Cas9^D10A^-EGFP. As shown in Figures 5B and C, the transfection resulted in the co-expression of Cas9^D10A^-EGFP and HSV1-tk-mCherry, indicating the insertion of HSV1-tk-mCherry into the target site. The insertion/expression rate of HUH7 cells reached 51.4% (Figure 5C). In contrast, the similar treatment of SNU449 cells, which were negative for these mutations, resulted in the expression of Cas9^D10A^-EGFP in most cells, but only 3.9% of cells expressed mCherry-HSV1-tk. These results suggested that the expression of HSV1-tk-mCherry was dependent on these specific mutations of SLTM. To investigate whether these mutation-targeting reagents had a therapeutic impact, SCID mice were xenografted with HUH7 cells. These mice were then treated with a cocktail of in vivo-JetPEI containing pCas9^D10A^-EGFP and pSLTM-mCherry-tk-gRNA 3 times a week after the tumors reached an average size of 114 mm^3^. As shown in Figure 5D, the treatment of these reagents resulted in smaller tumor volumes by 6-fold in comparison with pSLTM-mCherry-tk-gRNA control (*p* < 0.01), and 5.3-fold with Cas9^D10A^-EGFP only control (*p* < 0.01). There was no incidence of metastasis/invasion in the treatment group. In contrast, all mice treated with the control reagents had events of metastasis/invasion (*p* < 0.01, Figure 5E). All control-treated animals succumbed to the xenografted HUH7 tumors in 32 days after the xenografting, while all the mice treated with the correct combination of reagents survived beyond this period (*p* = 0.0018, Figure 5F). Taken together, these results suggest that genome targeting is effective in treating cancers that contain targetable mutations.

## DISCUSSION

Genome editing technology has been known for decades. However, the utility of genome editing technology was limited until precision editing was discovered in the CRISPR-Cas9 system. Previous studies have shown that chromosome rearrangement and point mutation in cancer were targetable through Cas9 editing.^14^,^39^ However, no study had been performed on single-nucleotide mutation targeting in liver cancer. In this report, we showed for the first time that the insertion of a suicide gene into a point mutation through Cas9 editing achieves effective cancer treatment in both spontaneous and xenografted liver cancer models. One of the mutations in our study occurred in CTNNB1, one of the most frequently mutated proto-oncogenes in liver cancer, while the other mutation occurred in SLTM, a regulator of RNA processing. Targeting at either mutation has achieved a high frequency of suicide gene insertion into the cancer genome and achieved partial remission of liver cancers that harbored these mutations. These studies may serve as a proof of principle in mutation targeting for human liver cancer.

The options for treating HCC of advanced stages remain few. Many small molecule treatments are minimally discriminatory toward normal and cancer cells. Thus, they may produce significant side effects. The Cas9-mediated insertion of a suicide gene such as HSV1-tk described in this study is mutation or chromosome rearrangement specific. In all 3 mutation/genome rearrangement targeting, the nonspecific expression of HSV1-tk in nontargeted cells was less than 5 percent. As a result, these treatments may have minimal impact on the healthy tissues. In general, mutations and chromosome rearrangement underlie the development of human cancers. The number of mutations and chromosome rearrangements may increase along the progression of liver cancer. Thus, the number of targetable mutations and chromosome rearrangements may increase. Flexibility in targeting may be required in controlling the progression of cancer. The versatility of Cas9-mediated mutation targeting may be well-suited for such a task. In clinical settings, the strategy described in this study can be utilized in the treatment of advanced liver cancer presented with targetable mutations after all other treatment options are exhausted.

The efficiency of suicide gene insertion into the mutation sites appeared variable, ranging from 54% to 98%, depending on the target site and cancer cells. The mechanism for such variability remains unclear. We speculate that the loci of the genome with active transcription may be more accessible to Cas9 nuclease and gRNA molecules due to the unwinding structure of the genome loci. In addition, the tertiary structure of the chromosome may impact the chromosome recombination that is required for the insertion of the suicide gene. Despite the high efficiency of insertion, the treatment did not completely eliminate the cancer in either spontaneous or xenografted cancer models, suggesting residual cancer may evolve to develop a mechanism to resist the single-target treatment. Further analysis is needed to understand the mechanism that evades the Cas9-mediated genome insertion of the suicide gene.

## Data Availability

Plasmid constructs will be available upon request. Jian-Hua Luo, Yan-Ping Yu, and Satdarshan P. Monga conceived the idea. Muhamuda Kader, Wei Sun, Junyan Tao, and Bao-Guo Ren performed the experiments. Jian-Hua Luo and Yan-Ping Yu supervised the project. Lesley M. Foley performed the imaging analyses. Silvia Liu performed the statistical study. All authors consent to publish.
